# Development of an Efficient *Agrobacterium*-Mediated Transformation Protocol for the Tetraploid Medicinal and Vegetable Species *Solanum villosum* Mill.

**DOI:** 10.3390/plants15203103

**Published:** 2026-10-10

**Authors:** Chun-Lan Piao, Min-Long Cui

**Affiliations:** Key Laboratory of Quality and Safety Control for Subtropical Fruit and Vegetable, Ministry of Agriculture and Rural Affairs, College of Horticulture, Zhejiang A&F University, Hangzhou 311300, China

**Keywords:** *Solanum villosum* Mill., tetraploid, medicinal and edible plant, in vitro shoot regeneration, *Agrobacterium*-mediated transformation, green fluorescent protein (GFP)

## Abstract

Genetic transformation is a cornerstone approach for functional genomics and molecular breeding in plants. *Solanum villosum* Mill. is a tetraploid species of the genus *Solanum* that possesses both nutritional and medicinal properties. However, its genetic improvement has been constrained by the lack of efficient transformation systems. In this study, a robust protocol was established for shoot regeneration and *Agrobacterium*-mediated transformation of *S. villosum* using leaf explants and *A. tumefaciens* strain EHA105/pBI35S::GFP, which harboring the Neomycin phosphotransferase II (*NPT II*) selectable marker and the green fluorescence protein (GFP) reporter gene. The results showed that 100% leaf explants produced adventitious shoots on semi-solid full-strength MS medium containing 1.0 mg L^−1^ thidiazuron (TDZ,) 0.1 mg L^−1^ α-naphthaleneacetic acid (NAA), 0.7% agar and 10 mg L^−1^ AgNO_3_, with an average of 7.8 shoots per explant after four weeks. Furthermore, transformation frequency was 81%, and over 90% of kanamycin-resistant plantlets showed a normal phenotype and stable GFP fluorescence. Moreover, transgene integration was verified by molecular analyses and fluorescence imaging of flowers, fruits, and T1 seedlings. Taken together, these results demonstrate that the tissue culture and transformation system established for *S. villosum* provides a valuable platform for large-scale propagation, gene function studies, and biotechnological improvement of this species.

## 1. Introduction

Plants in general serve as an indispensable reservoir of both nutrients and bioactive compounds, with approximately 80% of the global population depending on traditional medicinal plants for primary healthcare [1,2]. In Europe, over 40% of pharmaceutical products are derived from plant sources [3]. However, many medicinal species, including *Solanum villosum* Mill., are increasingly threatened by habitat degradation, climate change, overexploitation, and intrinsic genetic variability, all of which affect the consistency and yield of target metabolites [4,5,6,7,8]. These challenges are further compounded by the variability in bioactive compound accumulation across different populations and morphological types.

To address these constraints, the development of sustainable production systems is urgently required. In this context, efficient propagation methods, coupled with genetic transformation and genome editing technologies, offer promising strategies for enhancing and stabilizing the production of valuable phytochemicals in *S. villosum* and related medicinal species [9,10].

**Figure 1:**
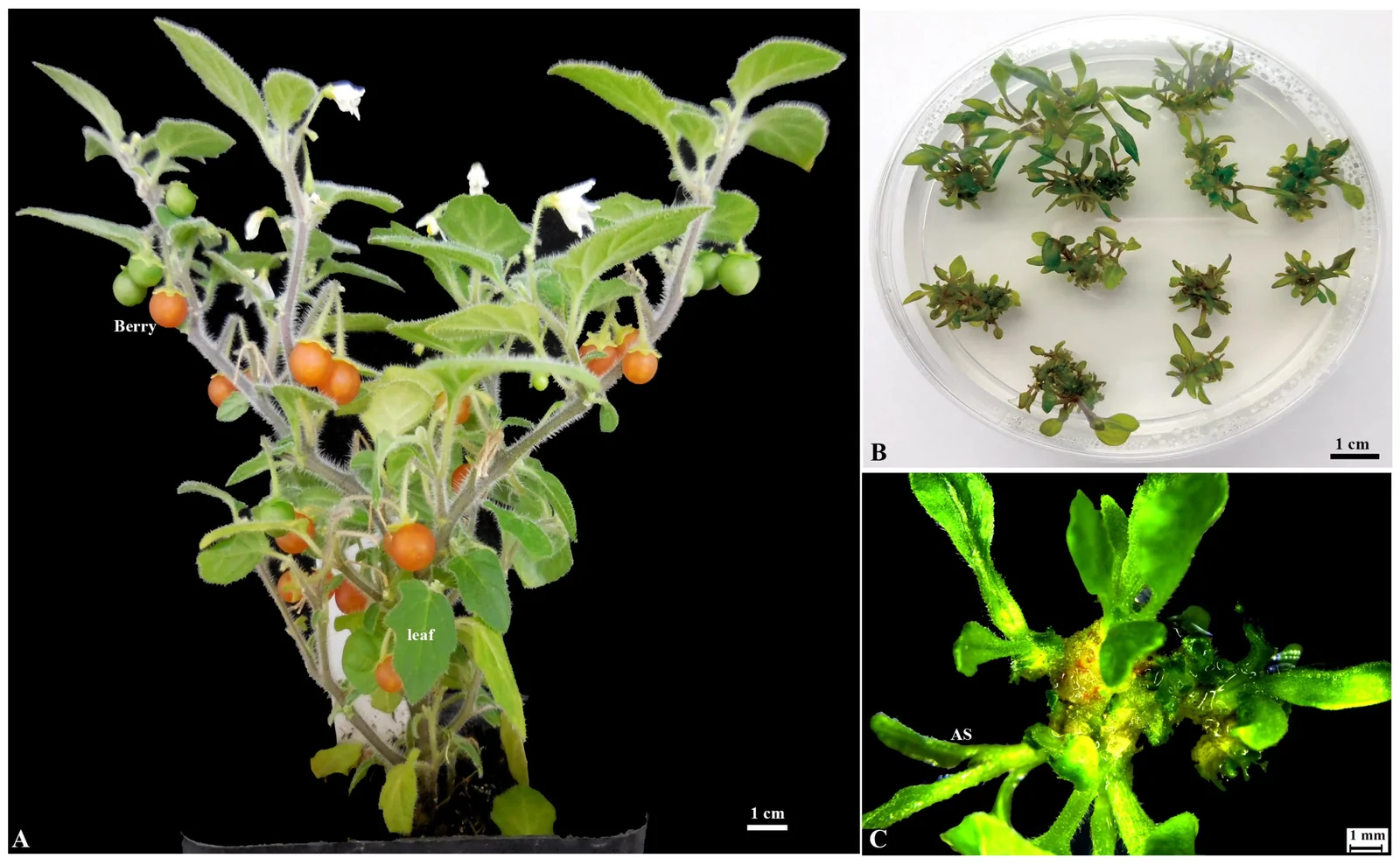
**Whole plant and shoot regeneration from leaf explants of** ***S. villosum*****.** (**A**) Two-month-old *S. villosum* grown in soil; (**B**) Shoot regeneration on semi-solid full-strength MS medium supplemented with 1.0 mg L^−1^ TDZ, 0.1 mg L^−1^ NAA, and 10 mg L^−1^ AgNO_3_ after 4 weeks of culture; (**C**) Close-up view of adventitious shoot regeneration from a leaf explant under the same conditions (**B**). AS: adventitious shoot.

*S. villosum* (Figure 1A), often referred to as the hairy nightshade [11], is a tetraploid species within the large and economically vital Solanaceae family [12,13]. Traditionally regarded as a cosmopolitan weed, it is increasingly recognized for its dual significance as both a nutritious leafy vegetable and a reservoir of medicinally active secondary metabolites [11,14,15]. This species is a rich source of vitamins, minerals, and health-promoting phytochemicals such as glycoalkaloids and phenolic compounds. It has long been used in traditional medicine to treat wounds, stomachache, tonsillitis, toothache, and hypertension-related fever, and is also consumed as a dietary component in parts of Africa and Asia [11,16]. Despite this notable potential, *S. villosum* has remained largely untouched by the biotechnological revolution that has profoundly reshaped research and breeding in other solanaceous crops. Although recent studies have reported well-established genetic transformation protocol for the closely related species *Solanum nigrum* L. (hexaploid) [17], significant evolutionary distance and genetic background divergence exist between *S. nigrum* and *S. villosum* (tetraploid) [12]. These two species also exhibit differential sensitivities to identical culture conditions, rendering the *S. nigrum* transformation methods not directly applicable to *S. villosum*. This technical gap constitutes a major bottleneck limiting functional genomics studies and trait dissection in this species. Therefore, it is imperative to develop a stable and reliable genetic transformation system for *S. villosum* to underpin subsequent investigations into gene functional analysis, metabolic pathway elucidation, and genetic improvement.

Plant transformation is an essential tool for functional genomics, genome editing, and molecular breeding. An optimal transformation system should be rapid, simple, and consistently reliable. Since its initial demonstration in tobacco [18], *Agrobacterium*-mediated transformation has been extensively employed in various crops for trait improvement and gene function studies [19,20,21]. Nonetheless, the efficiency of this approach is critically determined by the regeneration capacity of explants in vitro. Although morphogenic regulators such as WUSCHEL and BABY BOOM (BBM-WUS), and a GRF–GIF chimeric proteins have been applied to improve shoot regeneration in several monocots [22,23], the utility of this system is limited to species with embryogenic regeneration pathways and is often associated with abnormal shoot development [24]. Therefore, the development of efficient and broadly applicable shoot regeneration methods remains a key challenge for the widespread implementation of transformation and genome editing in plants.

Here, a stable and efficient *Agrobacterium tumefaciens*-mediated transformation protocol for *S. villosum* is presented. This method requires only a few fresh leaf explants and a tissue culture period of less than two months. The high efficiency and reliability of this protocol establish *S. villosum* as a versatile platform for gene functional characterization and regulatory mechanism dissection of secondary metabolic pathways.

## 2. Results

### 2.1. Optimization of Shoot Regeneration from S. villosum Leaf Explants

To determine the optimal conditions for stable shoot regeneration from *S. villosum* leaf explants, the effects of TDZ or BAP alone or in combination with 0.1 mg L^−1^ NAA and 10 mg L^−1^ AgNO_3_ were evaluated. After two weeks of culture, small green nodules emerged at the cut edges of the explants. After four weeks of culture, both BAP and TDZ alone resulted in low regeneration efficiency, among them 1.0 mg L^−1^ TDZ induced adventitious shoots in only 50% of explants (10/20), with an average of 1.55 ± 1.67 shoots per explant, and shoot elongation was slow (Table 1). In contrast, combinations of 0.1 mg L^−1^ NAA and TDZ markedly improved the frequency of shoot regeneration (Table 1). The combination of 0.1 mg L^−1^ NAA with 0.75 or 1.0 mg L^−1^ TDZ induced shoot formation in 100% of explants, yielding 6.5 and 7.8 shoots per explant, respectively (Figure 1B,C; Table 1). Notably, the highest shoot regeneration efficiency was achieved with the combination of 1.0 mg L^−1^ TDZ and 0.1 mg L^−1^ NAA, and all shoots were morphologically normal and grew vigorously (Figure 1B,C; Table 1). After five weeks of culture, morphologically normal and fast-elongating shoots were excised and transferred to semi-solid half-strength MS medium supplemented with 0.7% agar without plant growth regulators, where most plantlets developed healthy roots within four weeks. Based on these results, semi-solid full-strength MS medium supplemented with 1.0 mg L^−1^ TDZ, 0.1 mg L^−1^ NAA and 10 mg L^−1^ AgNO_3_ was selected for subsequent transformation experiments.

### 2.2. Kanamycin Sensitivity Assay

To evaluate the sensitivity of *S. villosum* leaf explants to kanamycin, twenty explants (approximately 0.6 × 0.6 cm^2^) per treatment were cultured on MS medium supplemented with 1.0 mg L^−1^ TDZ, 0.1 mg L^−1^ NAA, and 10 mg L^−1^ AgNO_3_. The medium contained kanamycin at concentrations ranging from 20 to 120 mg L^−1^, with a kanamycin-free medium serving as the control (Figure S1). The experiment was independently repeated three times. Explants grown on medium without kanamycin developed normally, with nearly all explants producing adventitious shoots. In contrast, kanamycin concentrations between 20 and 100 mg L^−1^ clearly inhibited explant growth. Notably, after 4 weeks of culture on medium containing 100 mg L^−1^ kanamycin, nearly almost explants (99.5%) turned brown or died (Figure S1). A complete mortality of explants was observed at 120 mg L^−1^ kanamycin treatment after 3 weeks of culture (Figure S1). Therefore, we determined that a concentration of 100 mg L^−1^ kanamycin was sufficient to inhibit both explant growth and shoot regeneration, and this concentration was used for subsequent transformation experiments.

### 2.3. Transformation of S. villosum

**Figure 2:**
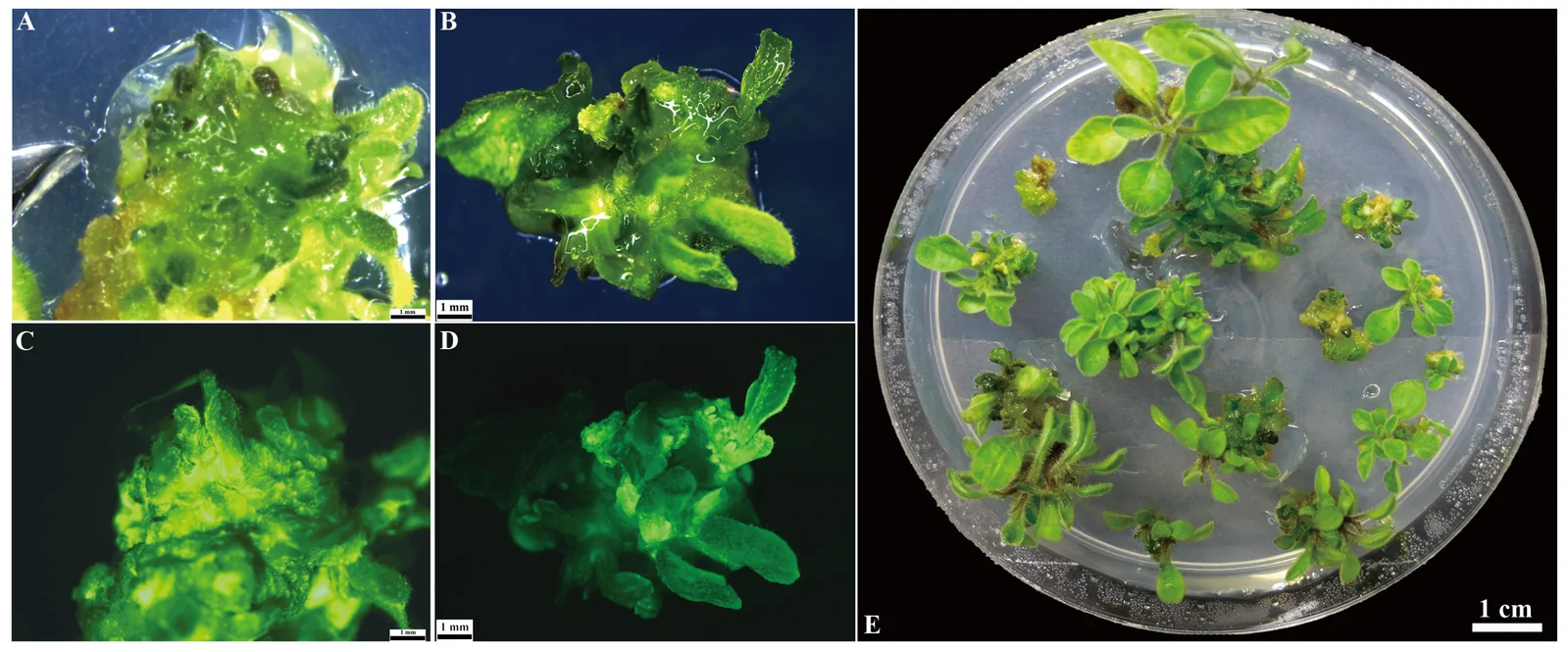
**Transformation process of** ***S. villosum*** **using leaf explants.** (**A**) Kanamycin-resistant shoots emerging the leaf explant after 3 weeks of culture on selection medium MS + 1.0 mg L^−1^ TDZ + 0.1 mg L^−1^ NAA +10 mg L^−1^ AgNO_3_ + 100 mg L^−1^ + kanamycin + 300 mg L^−1^ cefotaxime; (**B**) Kanamycin-resistant shoots forming from leaf explants on the same selection medium after 4 weeks culture; (**C**) GFP expression observed in the corresponding tissues (**A**); (**D**) GFP expression observed in the corresponding tissues (**B**); (**E**) Morphologically normal kanamycin-resistant shoots culture on the selection medium after 6 weeks.

**Figure 3:**
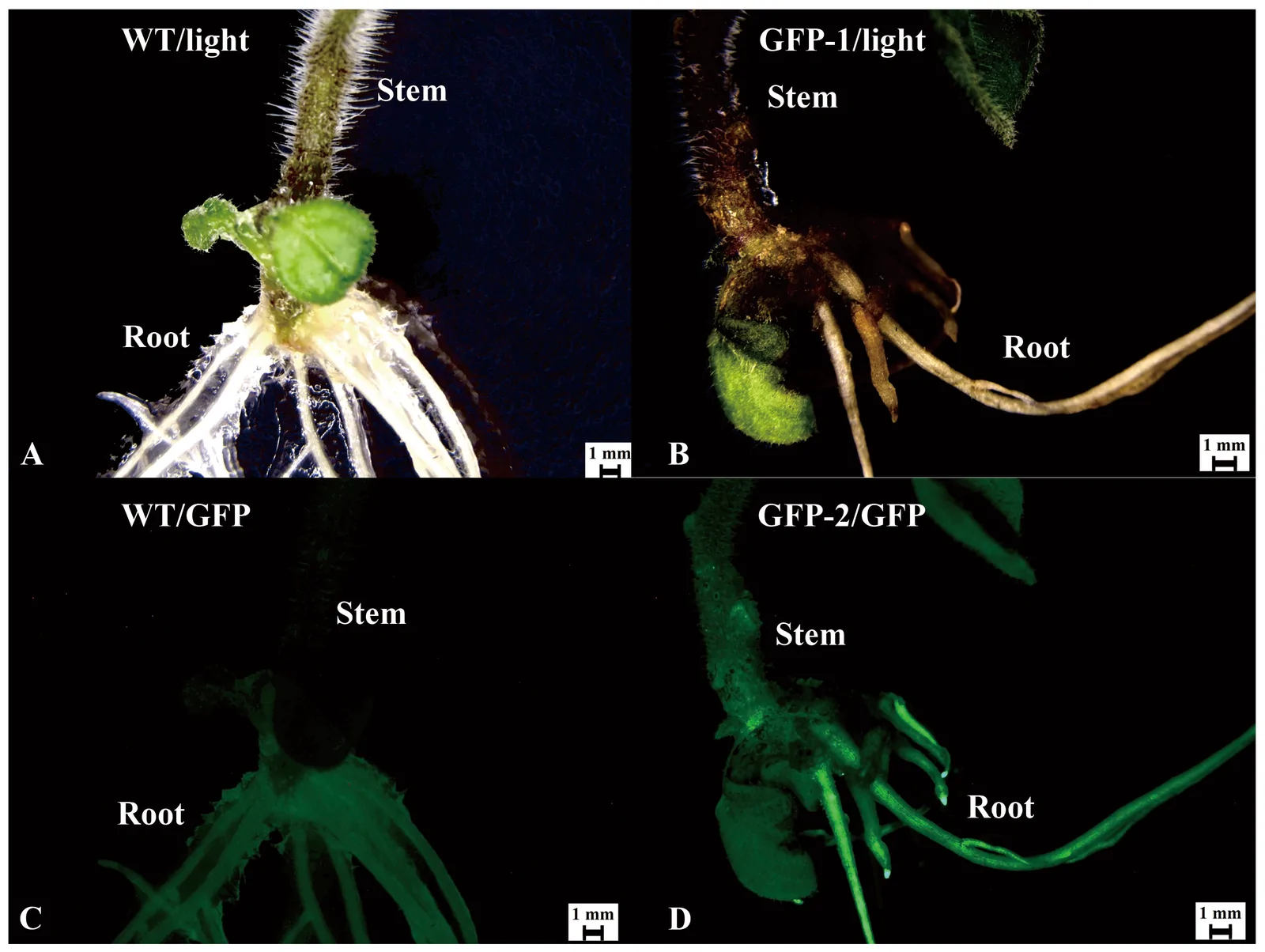
**Observation of GFP fluorescence in roots of transformed** ***S. villosum*****.** Fluorescence was observed using a Leica M165C fluorescence microscope. (**A**,**B**) Wild-type root under white light (**A**) and corresponding GFP fluorescence (**B**); (**C**,**D**) Kanamycin-resistant transgenic root under white light (**C**) and corresponding GFP fluorescence (**D**).

For transformation of *S. villosum*, leaf explants (n = 20 per set) were immersed in diluted *Agrobacterium* suspension, followed by co-cultivation on co-culture medium in the dark for three days, and the explants were then transferred to selection medium. After three weeks on selection medium, adventitious shoot emerged on compact green nodules exhibiting strong GFP fluorescence at the margins of the leaf explants (Figure 2A,C). After four weeks of culture on selection medium, numerous adventitious shoots had regenerated from these nodular structures (Figure 2B,D). After six weeks of culture, one morphologically normal and vigorously growing shoot per explant was excised and transferred to semi-solid 1/2MS medium supplemented with 50 mg L^−1^ kanamycin and 300 mg L^−1^ cefotaxime for inducted roots (Figure 2E). After 3–4 weeks on rooting medium, approximately 90% of kanamycin-resistant regenerated plantlets developed normally, and exhibited strong GFP expression (Table 2; Figure 3).

### 2.4. Molecular Analysis of Transformed S. villosum

**Figure 4:**
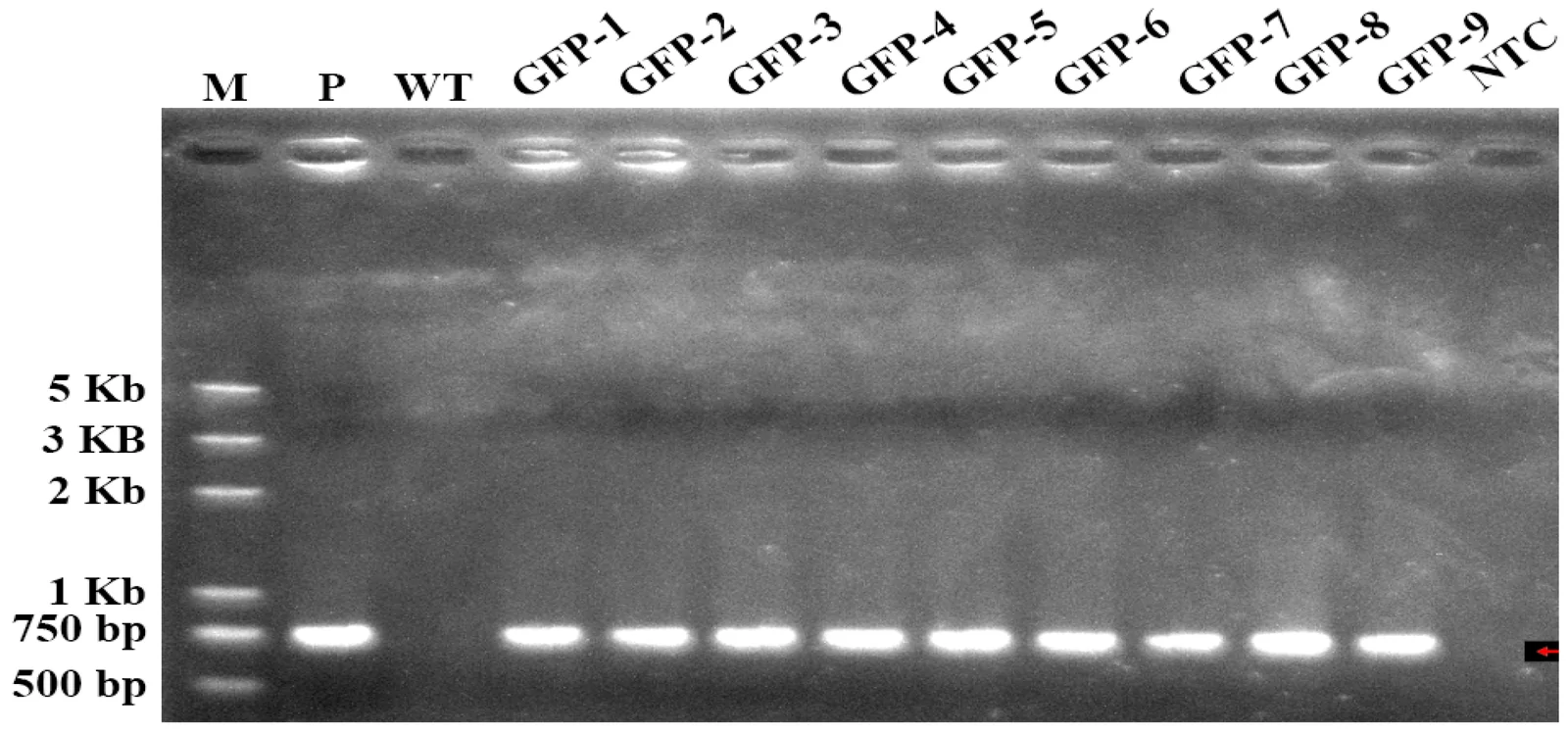
**PCR analysis of putative transgenic** ***S. villosum*** **plants using** ***GFP*****-specific primers.** Genomic DNA was extracted from young leaves of wild type (negative control) and nine independent putative transgenic lines that exhibited strong GFP fluorescence under a fluorescence microscope. M: 1 kb DNA Ladder marker (Takara, Tokyo, Japan); P: pBI-35S::GFP; WT: wild type plant; GFP-1 to -9: Nine independent GFP-positive lines; NTC: sterile water instead of template. The red arrow indicates the GFP-specific band.

*GFP* expression was detected in the roots of small plants four weeks after culture on rooting medium (Figure 3). The genomic integration of the *GFP* gene was confirmed by PCR in nine kanamycin-resistant lines (Figure 4). A specific band corresponding to the *GFP* gene was detected in the positive control pBI-35S::GFP and all nine transgenic lines (Figure 4, indicated by red arrow), whereas no amplified band was observed in either the non-transformed wild-type (WT) control or no-template control (NTC) (Figure 4). These results confirm the stable integration of the *GFP* gene in the transformed *S. villosum* plants.

### 2.5. Visualization of GFP Expression in S. villosum Development

**Figure 5:**
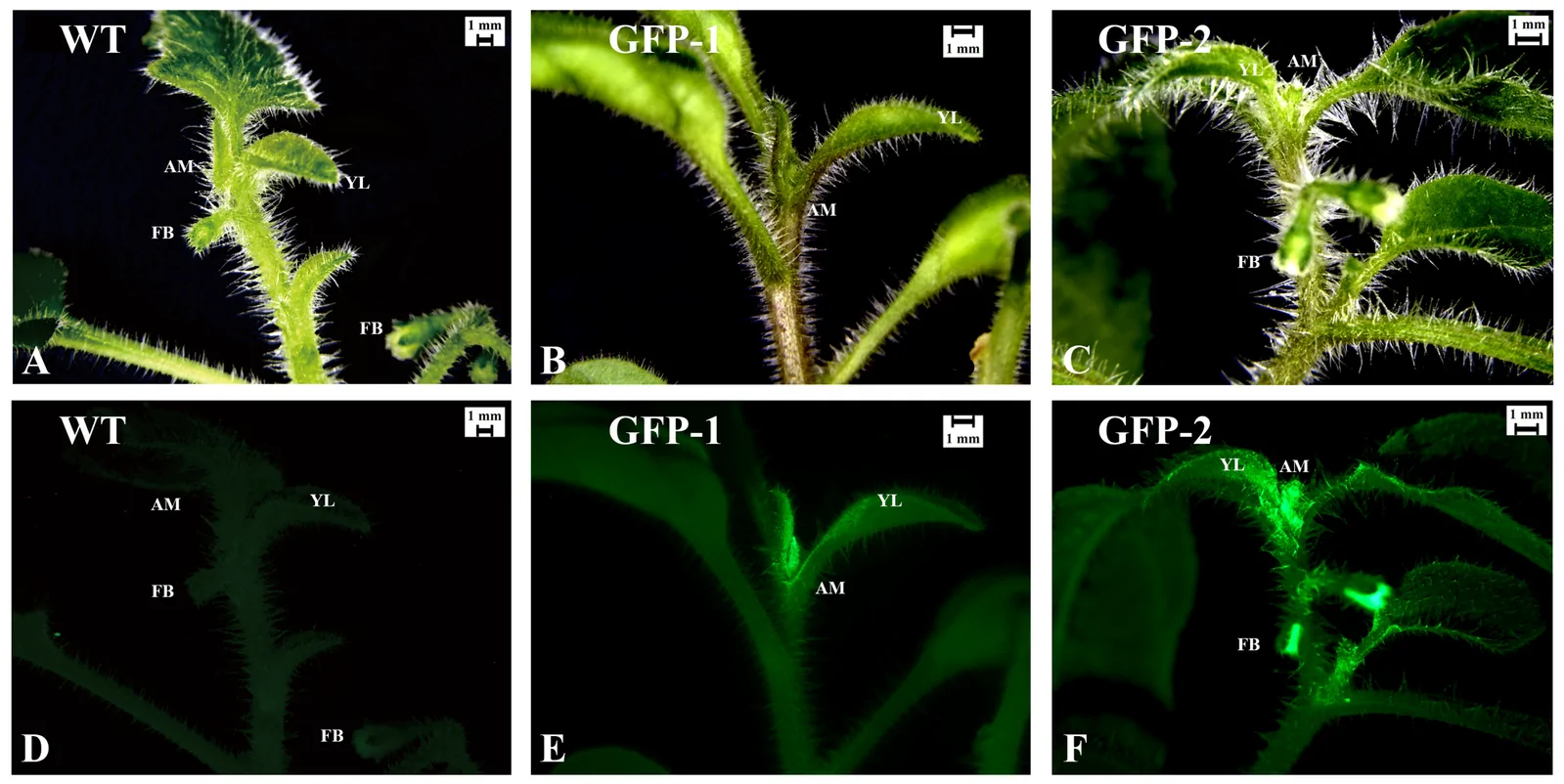
**Detection of GFP fluorescence in transgenic** ***S. villosum*** **plants.** (**A**–**C**) Bright-field images of wild-type (**A**), and transgenic lines GFP-1 (**B**) and GFP-2 (**C**); (**D**–**F**) Corresponding GFP fluorescence images of wild-type (**D**), and transgenic lines GFP-1 (**E**) and GFP-2 (**F**). Fluorescence signals were observed using a Leica M165C fluorescence microscope. YL, young leaf; AM: apical meristem; FB: floral bud.

**Figure 6:**
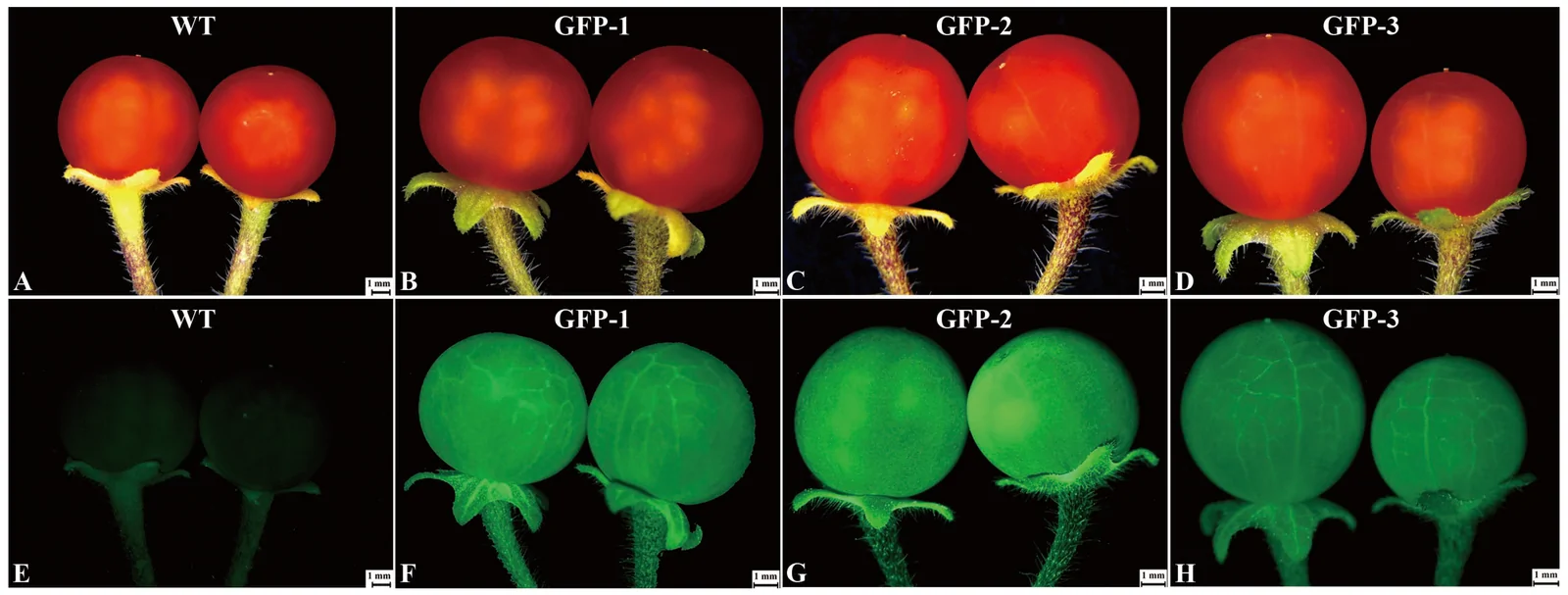
**Observation of GFP fluorescence in fruits of transgenic** ***S. villosum*****.** (**A**–**D**) Bright-field images: wild-type (**A**) and transgenic lines GFP-1 (**B**), GFP-2 (**C**), and GFP-3 (**D**); (**E**–**H**) Corresponding GFP fluorescence images: wild-type (**E**), GFP-1 (**F**), GFP-2 (**G**), and GFP-3 (**H**). Fluorescence signals were observed using a Leica M165C fluorescence microscope.

Stable GFP expression was observed in two transgenic lines across both vegetative and reproductive stages, including leaves, apical meristems, early floral buds, and mature fruits using fluorescence microscopy (Figure 5 and Figure 6). In the wild-type controls, no endogenous fluorescence was detected in leaves, apical meristem, flower buds or fruits (Figure 5A,D and Figure 6A,E), confirming the specificity of the GFP signal. In contrast, all nine transgenic lines displayed robust and uniform GFP fluorescence throughout the examined leaves, apical meristems, floral buds and fruits (Figure 5B,C,E,F and Figure 6B–D,F–H). These results indicate that the *GFP* transgene is stably expressed not only during vegetative growth stages but also in reproductive organs, suggesting that the transformation event did not disrupt during development process.

### 2.6. Observation of GFP Expression in T_1_ Progeny

**Figure 7:**
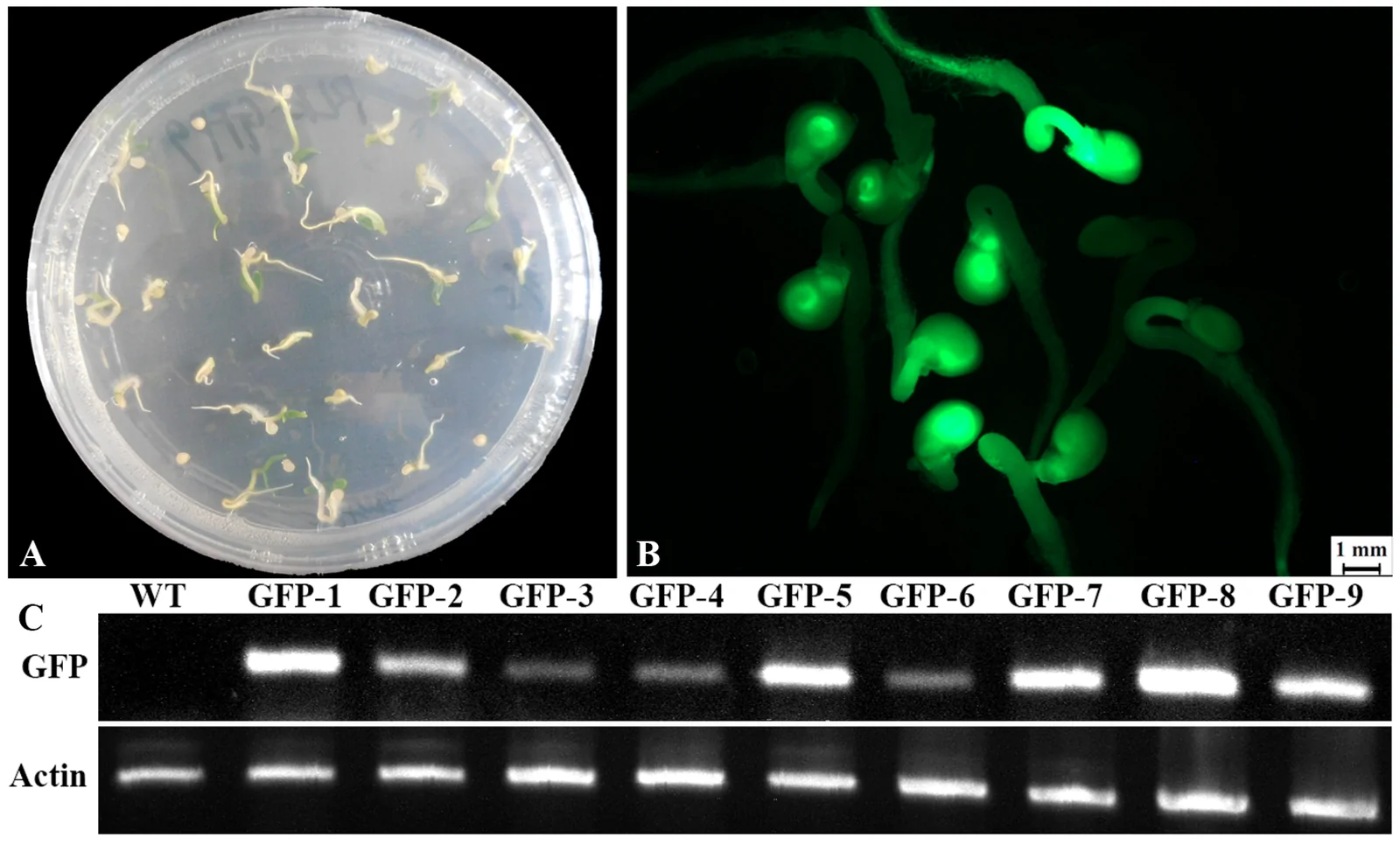
**GFP fluorescence and RT-PCR analysis in T_1_ progeny of transgenic** ***S. villosum*****.** (**A**) Representative image of 10-day-old seedlings of the GFP-1 transgenic line; (**B**) Observation of GFP fluorescence in the same seedlings under a fluorescence microscope; (**C**) RT-PCR analysis of *GFP* expression in T_1_ progeny. Total RNA was isolated from 2-week-old seedlings of wild-type (WT) and nine independent transgenic lines that exhibited strong GFP fluorescence. RT-PCR was performed using gene-specific primers for *GFP*, and *Actin* as an internal control. WT: wild-type; GFP-1 to -9: Nine independent transgenic lines.

Stable inheritance of *GFP* expression was confirmed in the self-pollinated T_1_ progeny of all nine transgenic lines (Figure 7). These seedlings germinated after 10 days after sowing (Figure 7A), and exhibited segregation between GFP-positive and GFP-negative individuals, whereas phenotypic traits including cotyledon size, hypocotyl length, and germination time showed no observable differences (Figure 7B). PCR analysis confirmed that GFP-positive seedlings carried the *NPTII* gene (Figure S2). Furthermore, to investigate *GFP* expression in T_1_ progeny, total RNA was extracted from 2 to 3 seedlings that showed strong GFP signals from each of the nine independent transgenic lines and analyzed for *GFP* expression by RT-PCR using *GFP*-specific primers. No *GFP* transcript was detected in wild-type seedlings (Figure 7C). In contrast, clear expression was detected in all nine independent transgenic lines (Figure 7C). These results suggest that the *GFP* gene was stably inherited and expression in the T_1_ progeny of transgenic *S. villosum*.

## 3. Discussion

Different plants or plant organs exhibit varying responses to the same phytohormones [25,26]. TDZ is a cytokinin that has been successfully used for shoot regeneration in *S. nigrum* [17], it is a closely related species with *S. villosum* in Solanaceae [12]. Therefore, the potential of TDZ for tissue culture of *S. villosum* was investigated in the present study. These combinations of TDZ and 0.1 mg L^−1^ NAA significantly enhanced shoot induction efficiency in leaf explants compared to TDZ alone (Table 1). Notably, leaf explants treated with 0.1 mg L^−1^ NAA combined with 0.75 or 1.0 mg L^−1^ TDZ yielded high frequencies of morphologically normal shoots (Figure 1B; Table 1). Ding et al. [17] previously reported that a 100% shoot induction rate from leaf explants of *S. nigrum* using 0.75 mg L^−1^ TDZ combined with 0.1 mg L^−1^ NAA [17]. They also reported that while 0.1 mg L^−1^ NAA combined with 1.0 mg L^−1^ TDZ promoted high shoot regeneration rates, this combination simultaneously enhanced callus formation and shoot vitrification [17]. In contrast, no obvious callus formation or shoot vitrification was observed under the same hormone combination in this study. This difference may be explained by the addition of 10 mg L^−1^ AgNO_3_, an ethylene inhibitor known to alleviate shoot vitrification, or alternatively, by species-specific variation in the endogenous hormone sensitivity of cytokinin receptors between the two *Solanum* species. In addition, only morphologically normal shoots were selected prior to rooting, which may have further limited the development of abnormal shoots and promoted rooting. Taken together, these findings suggest that shoot regeneration efficiency is highly sensitive to the combination of 1.0 mg L^−1^ TDZ with 0.1 mg L^−1^ NAA, and that 10 mg L^−1^ AgNO_3_ acts as a promoting factor facilitating the production of morphologically normal shoots from leaf explants of *S. villosum*.

Antibiotic- or herbicide-resistant genes are wildly used as selectable markers to facilitate the identification of transgenic events in plant transformation studies [27,28]. However, selective agents often inhibit cell proliferation and differentiation, thereby impairing adventitious shoot regeneration during tissue culture. Moreover, some plant species exhibit intrinsic insensitivity to these agents, which complicates the efficient discrimination between transformed and non-transformed cells, and increases the likelihood of false-positive escapes [29,30]. The neomycin phosphotransferase II gene (*NPTII*), which confers resistance to kanamycin and related aminoglycoside antibiotics, has been validated as a reliable selectable marker in various species, including peppermint [31], *Arabidopsis* [32], and kiwifruit [33]. However, its optimal concentration often requires species-specific calibration. For example, kanamycin at 50 mg L^−1^ effectively inhibits non-transformed *Arabidopsis* seedlings [32], whereas concentrations up to 200 mg L^−1^ have been successfully applied in peppermint without compromising transformation efficiency [31]. In this study, we observed that wild-type leaf explants of *S. villosum* displayed complete growth inhibition at 100 mg L^−1^ kanamycin, with 99% of the explants showing growth arrest or necrosis after four weeks of culture (Figure S1). Moreover, the use of an *NPTII* as a selectable marker revealed that over 90% of the kanamycin-resistant adventitious shoots exhibited robust *GFP* fluorescence. This finding suggests that 100 mg L^−1^ concentration could serve as a stringent selection threshold and *NPTII* is a suitable selectable marker for this species.

The choice of *Agrobacterium* strain is crucial for successful plant genetic transformation [17,34,35]. In this study, we tested the virulent strain EHA105 [36], which harbors the binary vector pBI-35S::GFP. This vector contains both the *NPTII* selectable marker gene and the *GFP* reporter gene. We achieved a transformation efficiency exceeding 81%, with approximately 90% of the kanamycin-resistant shoots exhibiting strong *GFP* expression two months post-inoculation (Figure 2; Table 2). Stable *GFP* expression was also observed in leaves, apical meristems, flowers, fruits, and their T_1_ generations (Figure 5, Figure 6 and Figure 7). These results demonstrate that EHA105 is an effective *Agrobacterium* strain for transforming *S. villosum* as well as the consistent expression across vegetative and reproductive organs further supports the reliability of the established transformation system.

## 4. Conclusions

In this study, we developed an efficient shoot regeneration method for tetraploid *S. villosum* using mature leaf explants cultured on semi-solid full-strength MS medium supplemented. Based on this method, we established a rapid and reliable *Agrobacterium*-mediated transformation system for *S. villosum*. Both the regeneration and transformation methods require only a small amount of leaf material and can be completed within two months. Notably, the transformation frequency exceeded 81%, and 90% of kanamycin-resistant plantlets exhibited normal growth and stable *GFP* expression throughout developmental stages and in their progeny. These simple and rapid shoot regeneration and transformation methods provide useful tools for micropropagation, functional gene research, elucidation of secondary metabolic regulatory pathways, production of valuable compounds such as steroidal alkaloids, flavonoids, and phenolic compounds, and genetic improvement of *S. villosum* via reverse genetics.

## 5. Materials and Methods

### 5.1. Seed Surface Sterilization and Stock Culture Establishment

Seeds of *Solanum villosum* Mill. were obtained from the seed stock maintained at Zhejiang A&F University. About 20 seeds were surface-sterilized with 70% ethanol for 30 s, then incubated in 0.1% (*w*/*v*) mercuric chloride solution for 3 min, followed by three times rinses with sterile distilled water. Sterilized seeds were germinated in Petri dish on semi-solid full-strength Murashige and Skoog (MS) [37] basal medium containing 1.5% sucrose and 0.7% agar without plant growth regulators, pH adjusted to 5.8 before autoclaving at 121 °C for 20 min. After two weeks of germination, seedlings were transferred individually to 200 mL glass vessels with 50 mL semi-solid half-strength MS (1/2 MS) medium containing 1.5% sucrose and 0.7% agar. For maintenance, ten shoot tips (2–3 cm) were excised and transferred to fresh semi-solid 1/2 MS medium containing 1.5% sucrose and 0.7% agar without plant growth regulators, every two months. All cultures were kept in a growth room at 25 °C under a 16/8 h light/dark cycle (100 μmol m^−2^ s^−1^). Fully expanded leaves from these plants were used as explants for tissue culture and transformation experiments.

### 5.2. Shoot Regeneration from Leaf Explant of S. villosum

To establishment suitable conditions for shoot regeneration from leaf explants of *S. villosum*, we evaluated a range of plant growth regulator combinations based on the regeneration method used for *S. nigrum* (Ding et al., 2023) [17]. Leaf explants were cut into approximately 0.6 × 0.6 cm^2^ pieces and cultured on semi-solid full-strength MS medium containing 3% sucrose, 10 mg L^−1^ AgNO_3_ (ethylene antagonist) and 0.7% agar supplemented with varying concentrations of thidiazuron (TDZ) or 6-benzylaminopurine (BAP) at 0.1, 0.25, 0.5, 0.75, and 1.0 mg L^−1^, either alone or in combination with 0.1 mg L^−1^ α-naphthaleneacetic acid (NAA). For each treatment, 20 explants were used per replicate, and the experiment was independently repeated three times. All cultures were maintained in a growth room under the same conditions as described above. After three weeks of cultivation, the enlarged explants were divided and sub-cultured in two identical Petri dishes containing fresh medium with the same concentrations of TDZ and NAA. Shoot regeneration frequency [Regeneration frequency (%) = No. of regenerating leaf explants/No. of leaf explants × 100%] was recorded after five weeks of culture and statistical analyses were performed using SPSS Statistics version 26.0.

### 5.3. Determination of Kanamycin Sensitivity in S. villosum Leaf Explants

To determine the sensitivity of *S. villosum* leaf explants to the selective agent kanamycin, leaf segments (0.6 × 0.6 cm^2^, n = 20 per treatment) were cultured on full-strength MS medium supplemented with 10 mg L^−1^ AgNO_3_ and varying concentrations of kanamycin (0, 20, 40, 60, 80, 100 and 120 mg L^−1^). The cultures were maintained in a growth room under the same conditions as described above. Each treatment was independently repeated three times, and the data are expressed as mean ± standard error (SE) of three biological replicates.

### 5.4. Arobacterium-Mediated Transformation of S. villosum

To develop a stable transformation protocol for *S. villosum*, we modified the *Agrobacterium*-mediated transformation method previously established for *S. nigrum* by Ding et al. [17]. The *Agrobacterium* strain EHA105, which harbored the pBI-35S::GFP vector [38], was used for all transformation experiments. Bacterial cultures were grown in 4 mL of liquid LB medium (1% peptone, 0.5% yeast extract, and 1% NaCl) supplemented with 50 mg L^−1^ kanamycin and 25 mg L^−1^ rifampicin at 28 °C for 20–24 h. The overnight culture was then diluted 1:20 with liquid MS basal medium containing 20 mg L^−1^ acetosyringone (AS) for OD_600_ =0.2~0.3. Fresh leaf explants (0.6 × 0.6 cm^2^, 20 per treatment) were immersed in the diluted bacterial suspension for 10 min, and then co-cultivated on semi-solid full-strength MS medium supplemented with 1.0 mg L^−1^ TDZ, 0.1 mg L^−1^ NAA, 0.7% agar and 20 mg L^−1^ AS at 25 °C in the dark for 3 days. The entire experiment was performed with three independent replicates. Following co-cultivation, the explants were transferred to selection medium [semi-solid full-strength MS medium supplemented 1.0 mg L^−1^ TDZ, 0.1 mg L^−1^ NAA, 0.7% agar, 10 mg L^−1^, AgNO_3_, 100 mg L^−1^ kanamycin and 300 mg L^−1^ cefotaxime] to eliminate *Agrobacterium* and select for transformed adventitious shoots. After five weeks of selection in a growth room, one morphologically normal and fast-growing kanamycin-resistant shoot was excised from each explant and transferred to rooting medium [semi-solid half-strength MS salts supplemented with 50 mg L^−1^ kanamycin and 300 mg L^−1^ cefotaxime] induced roots. After four weeks of culture on rooting medium, rooted plantlets were transferred to plastic pots (10 cm in diameter) containing a sterile mixture of peat moss and vermiculite 1:1 (*v*/*v*). The pots were covered with transparent plastic lids to maintain high humidity and acclimatized in a growth chamber at 25 °C under a 16 h light/8 h dark photoperiod. The lids were gradually removed over 7 days. The acclimatized plants were then maintained under the same conditions and used for further molecular characterization.

### 5.5. PCR Detection of Transgene S. villosum

Putative transgenic plants were preliminarily identified via polymerase chain reaction (PCR) analysis. Genomic DNA was extracted from leaves of kanamycin-resistant regenerants using the EasyPure^®^ Universal Plant Genomic DNA Kit (TransGen Biotech, Beijing, China). Genomic DNA from wild-type plants was used as a negative control, sterile water was used as a no-template control (NTC), and plasmid pBI-35S::GFP was used as a positive control. The independent primer sets were used for transgene detection: *GFP*-F (ATGGTGAGCAAGGGCGAGGAGC) and *GFP*-R (TTACTTGTACAGCTCGTCCATGC) for the *GFP* gene, *NPTII*-F (AGATGGATTGCACGCAGGTTC) and *NPTII*-R (GTGGTCGA ATGGGCAGGTAG) for the *NPTII* gene. Each PCR was performed in a total volume of 20 µL, containing 12.5 µL of Ex Taq PCR premix (Takara Bio, San Jose, CA, USA), 1 µL of template genomic DNA (50–100 ng µL^−1^), 1 µL of each primer at 10 µM, and 5.5 µL of nuclease-free double-distilled water. Thermal cycling conditions were as follows: initial denaturation at 96 °C for 3 min; 35 cycles of denaturation at 95 °C for 40 s, annealing at 58–60 °C for 40 s, and extension at 72 °C for 50 s; followed by a final extension at 72 °C for 10 min. Amplified products were separated on 1% (*w*/*v*) agarose gels and visualized under UV light. Each PCR was performed in triplicate, and all experiments were repeated three times using independently extracted DNA samples.

### 5.6. Observation of the GFP Expression During Transformation Process

To visually track transgene expression throughout the transformation process, we monitored GFP fluorescence at several key developmental stages: adventitious shoot initiation, shoot elongation, root formation, flowering, and fruit development as well as the T1 progeny. The fluorescence observation was conducted using a Leica M165 FC fluorescence microscope (Leica, Wetzlar, Germany) equipped with a GFP2 filter set (excitation 470/40 nm, emission 525/50 nm). Digital images were captured with a Leica DFC310 FX CCD camera and processed using LAS V4.2 imaging software (Leica, Wetzlar, Germany).

### 5.7. GFP-Expression Analysis by Reverse Transcription PCR

To confirm transgene expression at the transcriptional level, a semi-quantitative reverse transcription PCR (RT-PCR) was performed on nine independently derived *GFP*-positive transgenic lines, using wild-type plants as negative control. Total RNA was extracted from 100 mg of fully expanded young leaves using the SV Total RNA Isolation System (Promega, Singapore). First-strand cDNA was synthesized from 2 μg of total RNA with SuperScript III Reverse Transcriptase (Invitrogen, Waltham, MA, USA). PCR amplification was carried out with 100 ng of cDNA as a template, using gene-specific primer GFP-F (ATGGTGAGCAAGGGCGAGGAGC)/GFP-R (TTACTTGTACAGCTCGTCCATGC) for GFP gene. Because *S. nigrum* and *S. villosum* are closely related species, the *Actin* primer pair SnActin-F (CGCGCGTAC ACTGATGTATTCAA) and SnActin R (TACAAAGGGCAGGGACGTAGTCAA) of *S. nigrum* [17], was used to amplify the corresponding actin fragment in *S. villosum* for RT-PCR analysis. PCR reactions were performed on a GeneAmp PCR System 2700 under the following conditions: initial denaturation at 96 °C for 3 min; 30 cycles of 96 °C for 40 s, 62 °C for 40 s, and 72 °C for 50 s; and a final extension at 72 °C for 10 min. The amplified products were resolved on 1.2% (*w*/*v*) agarose gels by electrophoresis at 100 V for 30 min.

## Figures and Tables

**Table 1:** Effects of NAA (1-naphthaleneacetic acid) and TDZ (thidiazuron) concentrations on shoot induction from *Solanum villosum* leaf explants. Twenty leaf explants as a set were cultured at 25 °C under a 16 h light/8 h dark condition. A representative example of the experimental results is presented and the results were scored 5 weeks after culture.

TDZ (mg L^−1^)	NAA (mg L^−1^)	No. of Leaf Explants	No. of Regenerating Leaf Explants	Shoot Regeneration Frequency (%) ^a^	No of Shoots per Leaf Explants ^b^
0.1	0	20	2	10.0 ^c^	0.15 ± 0.277 ^c^
0.25	0	20	4	20.0 ^bc^	0.30 ± 0.248 ^c^
0.5	0	20	5	25.0 ^bc^	0.55 ± 0.378 ^c^
0.75	0	20	7	35.0 ^bc^	0.85 ± 0.472 ^bc^
1.0	0	20	10	50.0 ^b^	1.55 ± 0.374 ^bc^
0.1	0.1	20	11	55.0 ^b^	2.50 ± 0.546 ^bc^
0.25	0.1	20	15	75.0 ^ab^	3.15 ± 0.461 ^b^
0.5	0.1	20	18	90.0 ^a^	5.45 ± 0.351 ^ab^
0.75	0.1	20	20	100.0 ^a^	6.50 ± 0.539 ^a^
1.0	0.1	20	20	100.0 ^a^	7.81 ± 0.183 ^a^

ᵃ Different lowercase letters indicate significant differences among treatments (chi-square test followed by pairwise comparisons with Bonferroni correction, *p* < 0.05). ᵇ Data are presented as mean ± SE based on 20 explants per treatment. Different lowercase letters indicate significant differences among treatments (Kruskal–Wallis test followed by Dunn’s post hoc test with Bonferroni correction, *p* < 0.05). All analyses were performed using SPSS Statistics version 26.0.

**Table 2:** Transformation efficiency of *Agrobacterium* EHA105 harboring pBI-35S::GFP.

Experiments	No. of Leaf Explants	No. of Kanamycin-Resistant Shoots ^a^	No. of GFP-Positive Shoots (%) ^b^
1	20	17	16 (80.0)
2	20	19	16 (80.0)
3	20	18	17 (85.0)
Total	60	54	49 (81.7)

^a^ GFP fluorescence was examined using a Leica M165 FC fluorescence microscope after 4 weeks of culture on rooting medium. ^b^ Transformation frequency (%): No. of GFP-positive shoots/no. of leaf explants × 100%. Non-rooted shoots were no scored.

## Data Availability

All data generated or analyzed during this study are included in the article and its Supplementary Materials.

## Supplementary Materials

The following supporting information can be downloaded at: https://www.mdpi.com/article/10.3390/plants15203103/s1, Supplementary Figure S1. Effect of different concentrations of kanamycin on survival of leaf explants. Supplementary Figure S2. PCR detection of *NPTII* gene in *GFP*-positive T_1_ seedlings of transgenic *S. villosum* using *NPTII*-specific primers.
