# Supplementary material for: Development of an Efficient Agrobacterium-Mediated Transformation Protocol for the Tetraploid Medicinal and Vegetable Species Solanum villosum Mill
Source: Plants (Basel). 2026 Oct 10;15(20):3103. doi: 10.3390/plants15203103 (PMC13708614; doi:10.3390/plants15203103)
Supplement: Supplementary file 1 [file plants-15-03103-s001.zip › plants-4564932-supplementary.pdf]

Supplementary materials

Development of an efficient *Agrobacterium*-mediated transformation protocol for the tetraploid medicinal and vegetable species *Solanum villosum* Mill.

Chun-Lan Piao, Min-Long Cui *

Key Laboratory of Quality and Safety Control for Subtropical Fruit and Vegetable, Ministry of Agriculture and Rural Affairs, College of Horticulture, Zhejiang A&F University, Hangzhou 311300, China.

*** Correspondence:** Min-Long Cui,

E-mail: minlong.cui@zafu.edu.cn

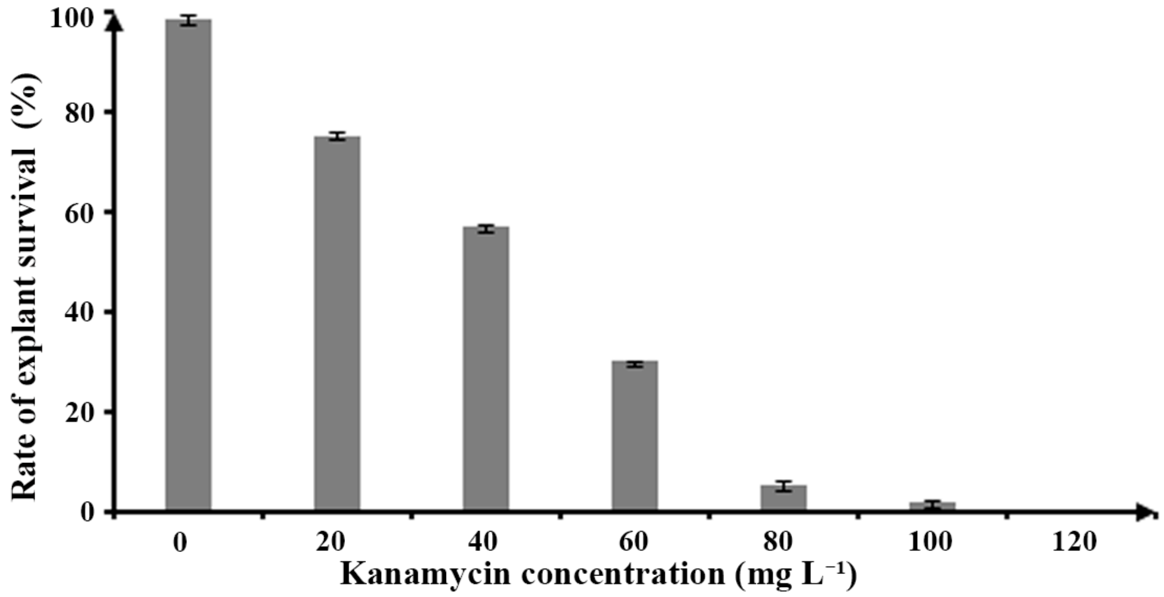

Supplementary Figure S1. Effect of different concentrations of kanamycin on survival of leaf explants. The percentage of normally growing explants (defined as explants showing normal growth, greenish callus, or visible shoot formation) was recorded after 4 weeks of culture. Each value represents the mean ± SE of three independent replicates (n = 20 explants per dish, 3 dishes per treatment).

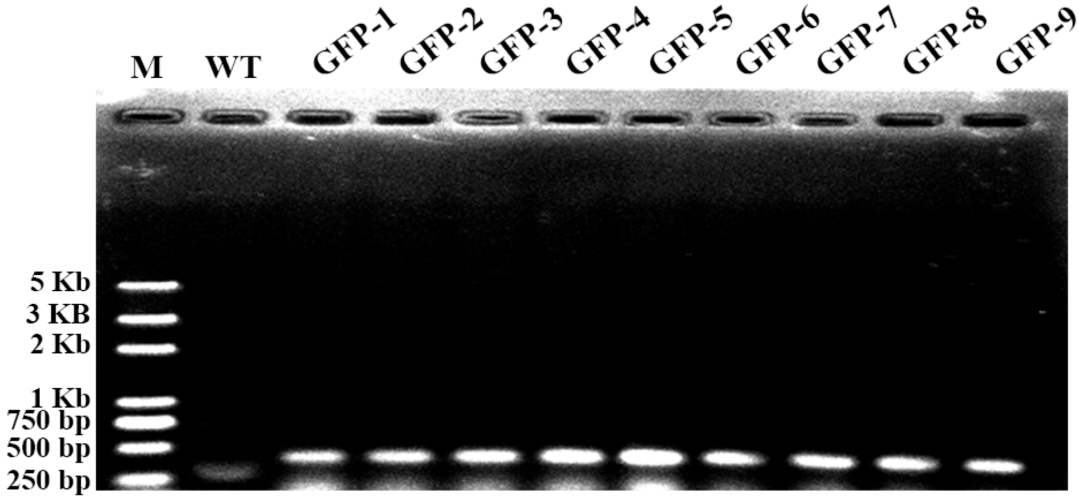

Supplementary Figure S2. PCR detection of *NPTII* gene in *GFP*-positive T_1 seedlings of transgenic *S. villosum* using *NPTII*-specific primers. Genomic DNA was extracted from 10-day-old wild-type seedlings (negative control) and nine independent transgenic lines with strong GFP fluorescence. M, 1 kb DNA ladder (Takara, Japan); WT: wild-type seedling; GFP-1 to -9: seedlings from nine independent GFP-positive lines.
